# Fish oil and probiotic food supplements: consumptions and attitudes of pregnant women in four European countries

**DOI:** 10.1007/s00394-025-03654-5

**Published:** 2025-04-05

**Authors:** Kristiina Jaakkola, Ella Koivuniemi, Kathryn Hart, Natalia Mazanowska, Romana Roccaldo, Laura Censi, Bernadette Egan, Lilja Mattila, Pasquale Buonocore, Eliisa Löyttyniemi, Monique Raats, Stefania Ruggeri, Miroslaw Wielgos, Kirsi Laitinen

**Affiliations:** 1 Integrative Physiology and Pharmacology Unit, Institute of Biomedicine, Faculty of Medicine, University of Turku, Turku, Finland; 2 Nutrition and Food Research Center, Faculty of Medicine, University of Turku, Turku, Finland; 3 Department of Nutritional Sciences, School of Biosciences and Medicine, Faculty of Health and Medical Sciences, University of Surrey, Guildford, UK; 4 Department of Obstetrics and Gynecology, Institute of Mother and Child, Warsaw, Poland; 5 Research Centre for Food and Nutrition, Council for Agricultural Research and Economics (CREA), Rome, Italy; 6 Biostatistics, Department of Clinical Medicine, University of Turku, Turku, Finland; 7 Department of Obstetrics and Perinatology, National Medical Institute of the Ministry of Interior and Administration, Warsaw, Poland; 8 Medical Faculty, Lazarski University, Warsaw, Poland; 9 Department of Obstetrics and Gynecology, Turku University Hospital, Turku, Finland

**Keywords:** Fish oil, Probiotics, Pregnancy, Recommendations

## Abstract

**Purpose:**

Fish oil and probiotic supplements may be of benefit during pregnancy, but no information on their use across geographically and socioeconomically diverse countries exists. Our aim was to investigate (1) usage of fish oil and probiotic food supplements by pregnant women, (2) awareness amongst pregnant women of the prevailing recommendations and (3) the characteristics of the users and their beliefs regarding potential health effects of food supplement use, and to compare these variables between women from four European countries.

**Methods:**

The survey was carried out by online questionnaires (*n* = 1780) in Finland (*n* = 536), Italy (*n* = 539), Poland (*n* = 584), and the United Kingdom (UK) (*n* = 121). Product information of the supplements used was collected from participants (*n* = 1356).

**Results:**

Of the participants 49% (*n* = 670) used fish oil, and 10% (*n* = 132) used probiotic supplements. The median intake of DHA in the studied countries was 220 (IQR 200–600) mg/d. Users of these supplements were most likely from Finland and primiparous. Recommendations related to fish oil supplement use were most well-known in Poland, where over half knew that fish oil supplements are recommended to be consumed during pregnancy in Poland. Finnish women were most likely to know that there is no recommendation for pregnant women for use of probiotic supplements.

**Conclusion:**

Half of the pregnant women used fish oil supplements, while probiotic use was less frequent. Not all pregnant women were familiar with the prevailing recommendations or potential health effects, which should be considered in the diet counselling provided during future health care visits.

**Supplementary Information:**

The online version contains supplementary material available at 10.1007/s00394-025-03654-5.

## Introduction

Food supplements are defined in Europe by the European Food Safety Authority (EFSA) as foods that are concentrated sources of nutrients, (e.g. vitamins or minerals), or other substances with a nutritional or physiological effect and are dosed e.g., in the form of tablets, capsules, or liquids [1]. Supplements are commonly used during pregnancy to support increased requirements due to maternal tissue growth and fetal growth and development [2], or to prevent potential pregnancy related problems including anaemia [3]. Some countries recommend the use of particular food supplements for pregnant women, most commonly iron and folic acid [3, 4].

Fatty fish is the major dietary source of omega-3 (n-3) long-chain polyunsaturated fatty acids (LC-PUFAs) which include docosahexaenoic acid (DHA) and eicosapentaenoic acid (EPA), the important fatty acids for fetal brain development and neurocognitive capabilities [5]. The amount of DHA that the human body can synthesize from its’ precursor, an essential fatty acid alpha linolenic acid (ALA), is insufficient considering the requirements of pregnancy and fetal and child development [6]. Therefore, maternal DHA intake from the diet is of importance and affects the amount that the fetus receives by placental transfer [7]. As fish consumption is variable [8], LC-PUFAs consumption as supplements may be beneficial. European food legislation permits the health benefits of LC-PUFA to be communicated to consumers as a health claims: ‘maternal DHA consumption contributes to normal brain and eye development in the fetus’ [9]. Further, some countries including Poland have included in their dietary recommendations for pregnant women to consume fish oil during pregnancy [10], whilst others, including Finland, Italy and the United Kingdom (UK), consider that the scientific evidence is insufficient to set a recommendation for this specific group of individuals. A daily intake of 200 mg DHA has been proposed to be required during pregnancy which may be achieved either by consuming fish or fish oil supplements [11].

Another food supplement that has raised interest through its potential health benefits if consumed during pregnancy are probiotics, defined by a consensus statement as “live microbes that, when administered in adequate amounts, confer a health benefit on the host” [12]. Consuming probiotics during pregnancy may reduce the risk of diseases in the offspring, such as eczema [13], as indicated by some randomized clinical trials [14, 15]. Further, mothers may benefit from consumption due to their anti-inflammatory potential which could reduce the risk of urogenital infections and mastitis [16]. Due to insufficient scientific evidence a general recommendation on the use of probiotics during pregnancy is not included in the guidance from many countries including those studied in this research (Finland, Italy, Poland, and the UK). The strongest evidence for the benefit of probiotic consumption during pregnancy relates to the lowered risk of childhood allergy and thus the World Allergy Organization (WAO) has issued an evidence-based recommendation for consumption of probiotics by allergy risk families [17]. It is of note that “probiotics” is considered a health claim in the European legislation, thus food supplement packages or marketing material may contain only the bacteria names as part of the ingredient list.

Although the research data on the health effects of the use of fish oil and probiotics during pregnancy is to some extent inadequate, and thus general recommendations for use during pregnancy have not been made, these food supplements are commercially available for consumers and no recommendation against their use during pregnancy has been made either. We have previously demonstrated that the use of multivitamin supplements is common, with 84% of the supplement users consuming these, during pregnancy [2]. The aim of this present study was to investigate (1) usage of fish oil and probiotic food supplements by pregnant women, (2) awareness amongst pregnant women of the prevailing recommendations and (3) the characteristics of the users and their beliefs regarding potential health effects of food supplement use, and to compare these variables between women from four European countries that are geographically and socioeconomically diverse: Finland, Italy, Poland, and the UK. This was carried out by survey and collection of information from food supplement product packages provided by the responders.

## Materials and methods

### Study design and population

Pregnant women were recruited through social media to participate in a cross-sectional survey on food supplements. The study information was targeted at women aged 18 to 45 years in Finland, Italy, Poland, and the UK and distributed via social media (Facebook) from July 2018 to June 2019. These countries were chosen because of the geographical and socio-economic differences, and because of the differences in the markets for food supplements. A target sample size of 500 participants per country was set, which is comparable to previous studies on food supplement use during pregnancy [18–20]. Additional recruitment channels including childbirth charity web pages were used in the UK. The criteria for inclusion were that the woman was pregnant and understood the main language of the home country. The study was conducted in accordance with the Declaration of Helsinki and approved by the Ethics committee for Human Sciences at the University of Turku, Turku, Finland for all countries (statement 63/2017). Additional ethical approval for Italy was received from the Ethics Committee of Lazio-2, Rome, Italy. Here we focus on the analysis of data relating to pregnancy use of probiotics and LC-PUFA (DHA + EPA), commonly known as fish oil, supplements, with data relating to other food supplements and to the requirements for breastfeeding provided for comparison only.

### Survey

Data collection, including information about the use of fish oil and probiotics supplements, knowledge of national recommendations, beliefs regarding the supplement use during pregnancy and participants characteristics, as well as product specific information, was conducted using an electronic questionnaire. Participant characteristics were collected and pre-pregnancy body mass index (BMI) [21] and physical activity level i.e. metabolic equivalent (MET) index [22] were calculated. Two pilot studies were conducted in each country before the main recruitment to test the feasibility of the recruitment method and the understandability of the survey.

Whilst filling in the survey, the responders were asked to add pictures of their food supplement packages, to add weblinks of the products or alternatively to describe the supplements they used (i.e. to provide product name, producer, trademark, nutritional content, and the dose taken). Based on the information provided, the daily intakes of fish oil, more specifically DHA and EPA, and probiotics were calculated, and the probiotic species used were specified. If the number of e.g. capsules was not reported by the women, the guide amount specified in the product information was used to calculate the intake, and if the guide amount was a range, e.g. 1–2, the smaller amount was used for the calculations. In addition, the frequency of fish oil and/or probiotic supplement use during pregnancy was requested (response options: I don’t use this supplement, options 1 to 7 days a week, less than 1 day a week, not sure).

**Fig. 1:**
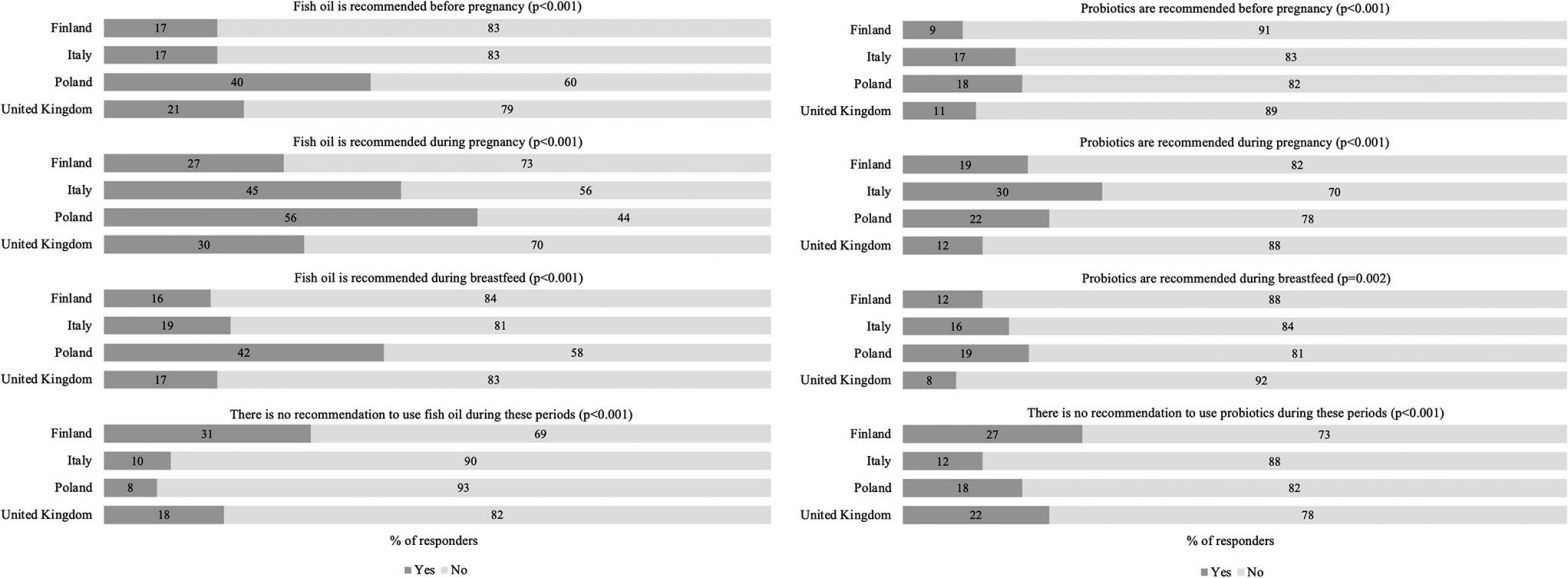
Participants (% of responders) awareness of the prevailing recommendations provided by governmental or national health bodies regarding the use of fish oil (on the left) and probiotic supplements (on the right) before and during pregnancy, and during breastfeeding (*n* = 1780), comparison between countries using Chi-square test

**Fig. 2:**
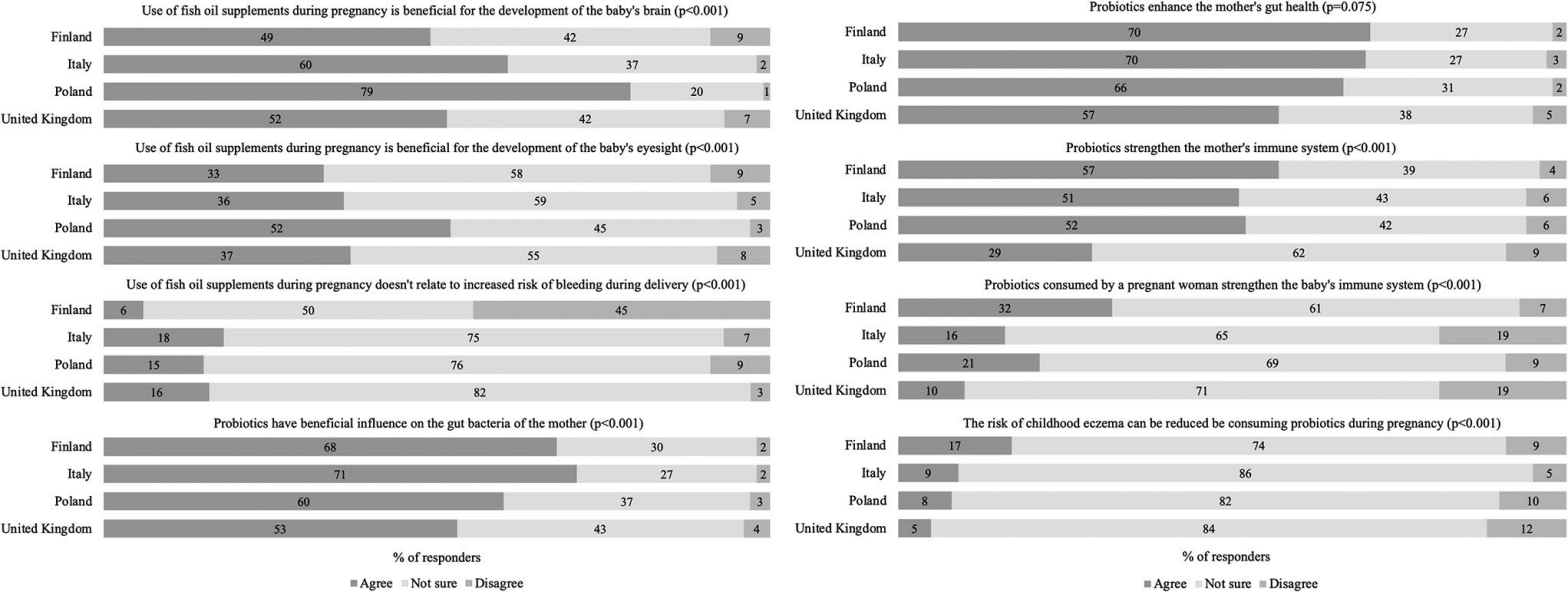
Participants (% of responders) beliefs about the potential health effects of fish oil and probiotic supplement use during pregnancy (total *n* = 1780, 6–14 participants missing from the analyses), comparison between countries using Chi-square test

Awareness of the prevailing recommendations provided by governmental or national health bodies regarding the use of fish oil and/or probiotics before and during pregnancy, and during breastfeeding was investigated by statements with response options: yes/no and I cannot say (statements listed in Fig. 1). The beliefs regarding the potential health effects of fish oil and probiotic supplement use during pregnancy were investigated by asking the participants’ level of agreement with statements with response options: strongly agree, agree, disagree, strongly disagree, not sure (statements listed in Fig. 2).

### Statistical analyses

Reported food supplements were included in the analysis if the woman had used them at least once a week during pregnancy. Comparison between countries was performed for nominal variables using the chi-squared or Fisher’s exact test, and for continuous data using the Kruskal-Wallis test. The figure on awareness of the recommendations (Fig. 1) did not take into account answers “I cannot say”. The response options “agree” and “strongly agree”, and the response options “disagree” and “strongly disagree” to the eight statements on fish oil or probiotics were combined for the figures, total *n* = 1780, 6–14 participants missing from the analyses (Fig. 2).

The statistical modelling for the use of fish oil and probiotics was conducted separately. The dependent variables, use of fish oil and probiotics, have three categories (no use of fish oil/probiotics, use of fish oil/probiotics, not using supplements at all). First, the association between each explanatory variable (country, parity, mother’s age, BMI, civil status, education, is mother working in health sector, smoking, alcohol use, MET index, does mother eat vegetables, fruit and berries, whole grain products or fish regularly) and use of fish oil/probiotics were analyzed with chi-square test or Fisher’s exact test. The chi-square test was also used to examine the relations between explanatory variables. If strong associations occurred between explanatory variables, only one was chosen to be entered into the multivariable modelling.

The multinomial logistic regression model for both dependent variables was built based on the results on a univariate approach (i.e. one explanatory variable at a time) explained above. Multivariable multinomial models were constructed including all significant explanatory variables from the univariate approach. The complete case analyses were performed using 1474 participants while all participants with missing data were automatically removed from the analyses. Full data of other food supplement users (i.e. prenatal multivitamin, iron, folic acid etc. vitamins and minerals), have been reported elsewhere [2]).

P-values (two-tailed) less than 0.05 were interpreted as statistically significant and the 95% confidence intervals were calculated. Statistical analyses were performed with SAS System version 9.4 (SAS Institute Inc., Cary, NC, USA) and SPSS version 27 (IBM Corp. Released 2020. IBM SPSS Statistics for Windows, Version 27.0. Armonk, NY: IBM Corp).

## Results

### Participant characteristics

In all, 1780 women were included in the analysis, 536 from Finland, 539 from Italy, 584 from Poland, and 121 from the UK (flowchart in the Online Resource 1). Of these 1356 participants provided product information for food supplements.

The demographic characteristics of the participants are shown in Table 1. The youngest participants were from Poland and the oldest from the UK and Italy. Two thirds of the participants were primipara. Based on BMI, 63% (1099/1758) had a healthy weight, whilst the highest percentages of participants with overweight or obesity were in Finland and the UK. Self-reported participant morbidity is listed in Online Resource 2. Most of the participants were well educated with university level education. Differences in smoking and drinking habits during pregnancy were observed between countries.

**Table 1:** Demographic characteristics of the participating women by country (total *n* = 1780)

	Total *n*^c^	All	Finland	Italy	Poland	The UK	*P*-value
**Maternal age**,** years**, median (IQR)	1778/535/539/583/121	30.0 (27.0–33.0)	30.0 (27.0–33.0)	32.0 (29.0–35.0)	28.0 (25.0–31.0)	32.0 (28.5–35.0)	< 0.001^b^
< 25 years, n (%)		187 (10.5)	45 (8.4)	21 (3.9)	105 (18.0)	16 (13.2)	< 0.001^a^
25–29 years, n (%)		657 (37.0)	217 (40.6)	140 (26.0)	280 (48.0)	20 (16.5)	
30–34 years, n (%)		630 (35.4)	201 (37.6)	219 (40.6)	162 (27.8)	48 (39.7)	
≥ 35 years, n (%)		304 (17.1)	72 (13.5)	159 (29.5)	36 (6.2)	37 (30.6)	
**Weeks of gestation**, median (IQR)	1779/536/538/584/121	24.7 (16.7–32.4)	24.4 (15.2–31.7)	24.4 (16.7–31.1)	25.6 (17.4–33.5)	25.1 (17.9–33.5)	0.016^b^
**Pre-pregnancy BMI**^**d**^, median (IQR)	1758/534/535/582/107	23.1 (20.7–26.3)	24.1 (21.5–27.7)	22.5 (20.4–25.5)	22.7 (20.5–25.5)	23.4 (21.5–27.1)	< 0.001^b^
Underweight, n (%)		101 (5.7)	11 (2.1)	39 (7.3)	48 (8.2)	3 (2.8)	< 0.001^a^
Normal weight, n (%)		1099 (62.5)	313 (58.6)	356 (66.5)	366 (62.9)	64 (59.8)	
Overweight, n (%)		361 (20.5)	112 (21.0)	93 (17.4)	132 (22.7)	24 (22.4)	
Obese, n (%)		197 (11.2)	98 (18.4)	47 (8.8)	36 (6.2)	16 (15.0)	
**Primipara**, n (%)	1779/535/539/584/121	1195 (67.2)	336 (62.8)	352 (65.3)	427 (73.1)	80 (66.1)	0.002^a^
**Diagnoses during this pregnancy**, n (%)	1776/536/538/581/121						
Gestational diabetes		142 (8.0)	62 (11.6)	25 (4.6)	52 (9.0)	3 (2.5)	< 0.001^a^
Pre-eclampsia and/or hypertension		29 (1.6)	7 (1.3)	10 (1.9)	12 (2.1)	0 (0.0)	0.360^a^
Pregnancy related nausea		628 (35.4)	213 (39.7)	206 (38.3)	157 (27.0)	52 (43.0)	< 0.001^a^
**Medical conditions**, n (%)	1747/536/528/565/118						
Allergy and/or asthma		340 (19.5)	170 (31.7)	67 (12.7)	86 (15.2)	17 (14.4)	< 0.001^a^
Thyroid disease		273 (15.6)	52 (9.7)	70 (13.39	141 (25.0)	10 (8.5)	< 0.001^a^
Migraine or frequent headaches		270 (15.5)	119 (22.2)	50 (9.5)	89 (15.8)	12 (10.2)	< 0.001^a^
Rheumatism, arthritis		29 (1.7)	5 (0.9)	12 (2.3)	9 (1.6)	3 (2.5)	0.317^a^
Heart disease/hypertension/high cholesterol		38 (2.2)	18 (3.4)	6 (1.1)	13 (2.3)	1 (0.8)	0.065^a^
Type I and/or type II diabetes							
Celiac disease and/or IBS^e^ and/or IBD^f^		17 (1.0)	7 (1.3)	5 (0.9)	5 (0.9)	0 (0.0)	0.609^a^
Food intolerance		148 (8.5)	50 (9.3)	46 (8.7)	32 (5.7)	20 (16.9)	< 0.001^a^
		111 (6.4)	44 (8.2)	42 (8.0)	16 (2.8)	9 (7.6)	< 0.001^a^
**Marital status**, n (%)	1730/513/534/570/113						< 0.001^a^
Living with a partner		611 (35.3)	227 (44.2)	222 (41.6)	132 (23.2)	30 (26.5)	
Married		1119 (64.7)	286 (55.8)	312 (58.4)	438 (76.8)	83 (73.5)	
**Mother’s education**, n (%)	1780/536/539/584/121						< 0.001^a^
Lower than university education		540 (30.3)	157 (29.3)	226 (41.9)	130 (22.3)	27 (22.3)	
University education		1240 (69.7)	379 (70.7)	313 (58.1)	454 (77.7)	94 (77.7)	
**Smoking regularly**, n (%)							
Before pregnancy	1778/535/539/583/121	549 (30.9)	108 (20.2)	239 (44.3)	182 (31.2)	20 (16.5)	< 0.001^a^
During pregnancy	1775/534/539/581/121	96 (5.4)	14 (2.6)	48 (8.9)	32 (5.5)	2 (1.7)	< 0.001^a^
**Alcohol use**,** at least sometimes**, n (%)							
Before pregnancy	1777/535/538/583/121	1422 (80.0)	448 (83.7)	388 (72.1)	478 (82.0)	108 (89.3)	< 0.001^a^
During pregnancy	1776/535/538/583/120	94 (5.3)	8 (1.5)	67 (12.5)	4 (0.7)	15 (12.5)	< 0.001^a^
**Leisure time physical activity, categorized MET index value**^**g**^, n (%)	1760/531/531/577/121						< 0.001^a^
Low activity		1247 (70.9)	268 (50.5)	440 (82.9)	476 (82.5)	63 (52.1)	
Moderate activity		465 (26.4)	224 (42.2)	87 (16.4)	98 (17.0)	56 (46.3)	
High activity		48 (2.7)	39 (7.3)	4 (0.8)	3 (0.5)	2 (1.7)	
**Working in health sector**, n (%)	1698/502/505/572/119	476 (28.0)	229 (45.6)	119 (23.6)	88 (15.4)	40 (33.6)	< 0.001^a^
**Diet**							
Eats fish at least once a week, n (%)	1776/535/538/583/120	948 (53.4)	276 (51.6)	367 (68.2)	241 (41.3)	64 (53.3)	< 0.001^a^
Uses vegetables twice a day, n (%)	1779/536/538/584/121	773 (43.5)	315 (58.8)	113 (21.0)	277 (47.4)	68 (56.2)	< 0.001^a^
Uses whole-grain products daily, n (%)	1777/535/539/583/120	973 (54.8)	420 (78.5)	180 (33.4)	296 (50.8)	77 (64.2)	< 0.001^a^

^a^Comparison between countries, Chi-square test

^b^Comparison between countries, Kruskal-Wallis test

^c^Total number of included participants is 1780, 1–82 participants missing from the analyses

^d^BMI categorized as underweight (BMI < 18.5), normal weight (BMI 18.5–24.9), overweight (BMI ≥ 25.0 and < 30.0) and obese (BMI ≥ 30.0)

^e^Irritable bowel syndrome

^f^Inflammatory bowel diseases (Crohn’s disease, Ulcerative colitis)

^g^MET index scores < 5 indicating light, scores 5–30 moderate and scores > 30 high physical activity

### Use of fish oil and probiotic supplements

Overall 49% (670/1356) of the participants from all the countries studied reported having used a food supplement containing fish oil and 10% (132/1356) had used probiotics during pregnancy (Table 2), most commonly daily for fish oil (daily users 62% in Finland, 74% in Italy, 83% in Poland and 68% in the UK) and for probiotics (daily users 54% in Finland, 47% in Italy, 52% in Poland and 57% in the UK).

When inspecting the data from the product information (Table 2), the largest percentage of fish oil users, 77% (336/436), was in Poland, followed by Italy and the UK, whilst Finland had the lowest percentage of users. The median intake of DHA from the supplements was 220.0 mg/d, and 70.0 mg for EPA/d. Participants from Poland had the highest intake of DHA, whilst Italy had the lowest.

The use of probiotic food supplements also differed between countries, Finland had the highest percentages of probiotic users, 18% (85/470), followed by the UK and Italy, whilst Poland had the lowest, 3% (11/436) (Table 2). *Lacticaseibacillus rhamnosus* (formerly *Lactobacillus rhamnosus*) was the most common species in the food supplements consumed in Finland and in Poland, while *Bifidobacterium lactis* in Italy, and both *Bifidobacterium lactis* and *L. acidophilus* were common in the UK. All the species itemized from the reported product information are listed in the Online Resource 3.

**Table 2:** Frequency of fish oil and probiotic supplement users, calculated intakes of DHA and/or EPA (mg/d), and the most common probiotic species from participants’ self-reported product information by country

	Total	All	Finland	Italy	Poland	United Kingdom	*P*-value
**Fish oil**, n (%)	1356/470/353/436/97	670 (49.4)	96 (20.4)	205 (58.1)	336 (77.1)	33 (34.0)	< 0.001^a^
**DHA intake** (mg/d), median (IQR)	667/93/205/336/33	220.0 (200.0-600.0)	272.0 (136.0-435.0)	200.0 (200.0-200.0)	500.0 (200.0-600.0)	300.0 (300.0-300.0)	< 0.001^b^
**EPA intake** (mg/d), median (IQR)	355/88/82/154/31	70.0 (40.0-120.0)	171.4 (73.6–360.0)	40.0 (40.0–40.0)	70.0 (70.0-71.3)	60.0 (60.0–64.0)	< 0.001^b^
**Probiotic users,** n (%)	1356/470/353/436/97	132 (9.7)	85 (18.1)	27 (7.6)	11 (2.5)	9 (9.3)	< 0.001^a^
**Most used probiotic species**^**d**^, n (%)							
*Lacticaseibacillus rhamnosus*	1356/470/353/436/97	87 (6.4)	70 (14.9)	8 (2.3)	6 (1.4)	3 (3.1)	< 0.001^a^
*Bifidobacterium lactis*	1356/470/353/436/97	66 (4.9)	47 (10.0)	12 (3.4)	2 (0.5)	5 (5.2)	< 0.001^c^
*L. acidophilus*	1356/470/353/436/97	57 (4.2)	44 (9.4)	5 (1.4)	3 (0.7)	5 (5.2)	< 0.001^c^
*L. casei*	1356/470/353/436/97	38 (2.8)	30 (6.4)	5 (1.4)	2 (0.5)	1 (1.0)	< 0.001^c^

^a^Comparison between countries, Chi-square test

^b^Comparison between countries, Kruskal-Wallis test

^c^Comparison between countries, Fisher’s exact test

^d^To shorten the text the top three most used probiotic species by country listed in Online Recourse 3

### Awareness of the supplement recommendations

Overall, participants from all countries studied had uncertainties about the recommendations for use (Fig. 1). In general participants thought that these food supplements are recommended for use during pregnancy, although Poland was the only country which had issued this recommendation for fish oil.

The largest proportion of the participants who were aware of the prevailing recommendation was in Poland where over half (328/584) of the women knew that fish oil supplements are recommended during pregnancy, while 31% (168/536) in Finland followed by 18% (22/121) in UK and 10% (52/539) in Italy knew that there is no recommendation to use fish oil during these periods. However, 27% (145/536) of the participants in Finland, 45% (240/539) in Italy and 30% (36/121) in UK mistakenly thought that it is recommended to use fish oil supplements during pregnancy.

There is no recommendation to use probiotic supplement in any of the countries, which was known by 27% (143/536) of the participants in Finland, 12% (65/539) in Italy, 18% (106/584) in Poland and 22% (27/121) in UK.

### Beliefs of the health effects

Participants’ beliefs regarding the potential health effects of the two supplements were explored with statements presented in a form that corresponds to the correct answer based on the scientific literature. Participants’ knowledge of the health effects of fish oil and probiotic food supplements varied with statistically significant differences between the countries (Fig. 2). The highest proportion of participants agreeing with the statements that fish oil is beneficial for the baby’s brain and eyesight was in Poland, while the lowest was in Finland. In Finland 45% (239/536) of the participants disagreed incorrectly with the statement that fish oil does not relate to an increased risk of bleeding during delivery, whilst in other countries most of the participants responded unsure. Over half of the participants from all the countries agreed that probiotics have beneficial influence on the gut bacteria of the mother, enhance the mothers gut health, and strengthen the mother’s immune system, except in the UK where less than one-third agreed with this statement. In Finland, the highest proportion of the participants agreed with the statements regarding the effects of probiotics on the offspring’s health, less than 10% of the participants from Italy, Poland, and the UK agreed that the risk of childhood eczema can be reduced by consuming probiotics during pregnancy.

### Characteristics of fish oil and other food supplement users

The characterization of fish oil supplement users and other food supplement users (i.e. prenatal multivitamin, iron, folic acid etc. vitamins and minerals) as compared to supplement non-users is presented in Table 3.

Participants who used fish oil supplement were most likely to be from Finland. Primipara women were more likely to be fish oil users than supplement non-users. Fish oil users’ pre-pregnancy BMI and habits of eating vegetables or using whole-grain products did not differ significantly to those not using supplements.

**Table 3:** Modelling the characteristics of selected sociodemographic and behavioral factors in fish oil supplement users (*n* = 664/1474) and other food supplement users (*n* = 678/1474) as compared to food supplement non-users (*n* = 132/1474)

	Fish oil food supplement user		Other food supplement^b^ user		
	Adjusted OR	95% confidence interval	Adjusted OR	95% confidence interval	Overall p-value^a^
**Home country**					< 0.0001
Finland	1		1		
Italy	0.18	0.08–0.42	0.03	0.01–0.07	
Poland	0.63	0.27–1.49	0.05	0.02–0.11	
United Kingdom	0.22	0.07–0.64	0.12	0.04–0.32	
**Pre-pregnancy BMI**					0.8739
Underweight / normal weight	1		1		
Overweight	0.83	0.51–1.33	0.85	0.52–1.40	
Obese	1.04	0.50–2.16	1.2	0.58–2.50	
**Primipara**					0.0009
Yes	1		1		
No	0.48	0.32–0.70	0.57	0.38–0.86	
**Use vegetables twice a day**					0.7501
Yes	1		1		
No	0.84	0.54–1.31	0.86	0.54–1.36	
**Use whole-grain products daily**					0.5021
Yes	1		1		
No	1.18	0.78–1.79	1.29	0.84-2.00	

^a^Overall p-values in multinominal logistic regression model including selected sociodemographic and behavioural factors

^b^Prenatal multivitamin supplements, other multivitamin supplements and supplements containing vitamin A, folate, vitamin B12, other B vitamins, vitamin C, vitamin D, vitamin E, iodine, calcium, iron, zinc, selenium, chromium, manganese, magnesium, and others of which the participants specified in writing

### Characteristics of probiotic and other food supplement users

The characterization of probiotic supplement users and other food supplement users (i.e. prenatal multivitamin, iron, folic acid etc. vitamins and minerals) as compared to supplement non-users is presented in Table 4.

Probiotic supplement users were more likely to be from Finland, and primiparous participants were more likely probiotic users than supplement non-users. Pre-pregnancy BMI and women’s habits of eating vegetables or using whole-grain products did not differ significantly when probiotic users were compared to supplement non-users.

**Table 4:** Modelling the characteristics of selected sociodemographic and behavioral factors in probiotic supplement users (*n* = 130/1474) and other supplement users (*n* = 1212/1474) as compared to supplement non-users (*n* = 132/1474)

	Probiotic food supplement user		Other food supplement^b^ user		
	Adjusted OR	95% confidence interval	Adjusted OR	95% confidence interval	Overall p-value^a^
**Home country**					< 0.0001
Finland	1		1		
Italy	0.03	0.01–0.08	0.07	0.03–0.16	
Poland	0.02	0.01–0.07	0.2	0.09–0.45	
United Kingdom	0.07	0.02–0.23	0.15	0.05–0.42	
**Pre-pregnancy BMI**					0.4791
Underweight / normal weight	1		1		
Overweight	0.98	0.52–1.82	0.83	0.52–1.31	
Obese	0.75	0.30–1.88	1.14	0.57–2.31	
**Primipara**					0.0022
Yes	1		1		
No	0.49	0.29–0.83	0.52	0.35–0.75	
**Use vegetables twice a day**					0.0759
Yes	1		1		
No	0.56	0.31–0.99	0.88	0.57–1.35	
**Use whole-grain products daily**					0.6204
Yes	1		1		
No	1.22	0.68–2.19	1.22	0.82–1.83	

^a^Overall p-values in multinominal logistic regression model including selected sociodemographic and behavioural factors

^b^Prenatal multivitamin supplements, other multivitamin supplements and supplements containing vitamin A, folate, vitamin B12, other B vitamins, vitamin C, vitamin D, vitamin E, iodine, calcium, iron, zinc, selenium, chromium, manganese, magnesium, and others of which the participants specified in writing

## Discussion

This study demonstrated that the use of fish oil and probiotic supplements during pregnancy differ between four European countries studied: Finland, Italy, Poland, and the UK. Overall, use of probiotic supplements was less common than that of fish oil supplements. There was uncertainty about the prevailing recommendations of the supplement use by the pregnant women in each country, as well as the health effects of the food supplements for pregnant women or for their unborn child.

In our study about half of the women reported using fish oil supplements and a tenth probiotic supplements during pregnancy. For fish oil these numbers indicate more frequent use than reported in the previous studies in which 4 to 16% of pregnant women in Denmark, Germany, Sweden, Australia and the United States had used the supplements [20, 25–26], but in Iceland the intake was similar, up to 50% among pregnant women [27], as in our study. The use of probiotic supplements during pregnancy has been 9% in other study [28], which is similar to our study. When we further characterized the users, both fish oil and probiotic users were most likely primipara, which is in line with previous findings [29–30, 26], in addition pregnant women use food supplements more often than non-pregnant women [31]. It is of note that our study focused on food supplement use and therefore may have been open to selection bias such that those women with an interest in supplementation were more likely to participate.

The median daily intake of DHA in the supplement consumers was in line with the consensus recommendation of 200 mg/d in each of the studied country (84% of all participants received 200 mg or more of DHA per day from the food supplements). It is of note that the consensus recommendation concerns intake from diet and from the supplements [11], whilst we had no information on the dietary intake of DHA. Nevertheless, intake of DHA from the supplements appears to be of importance as the intake from diet is typically insufficient: e.g. in a previous Polish study only 8% (*n* = 100) of the pregnant women had intake > 200 mg/d DHA from food (mainly fish and eggs), and only 10% of the pregnant women ate fish twice a week as recommended [32]. It was shown in a cross-sectional survey that pregnant women in the US had a mean intake of 67.5 mg/d of DHA from the food [24]. In our study Poland had the highest intakes of DHA from the supplements and the highest percentage of participants who used fish oil supplements compared to other countries studied. This is reasonable as Poland is the only country with a recommendation to use DHA as a food supplement (200 mg/d for all, and 1000 mg/d for those at risk) during pregnancy [10]. The knowledge of the prevailing recommendations varied in each country, the recommendations were most familiar in Poland where 56% knew about the recommendation to use fish oil during pregnancy, while 31% in Finland knew correctly that there is no recommendation for fish oil usage during these periods. Therefore, diet counselling is needed during health care visits to achieve sufficient intake, e.g. for DHA, which is important for fetal development. However, it has also been noticed that for some food supplements, the daily safe upper intake limit has been exceeded in pregnant women, for e.g. magnesium, folic acid [2] and Fe [33], which emphasizes the importance of instructions for proper use.

In comparison between the four countries, the consumption of probiotics supplements was most common in Finland. The most commonly used probiotic species was *Lacticaseibacillus rhamnosus* whilst the second most common species was *Bifidobacterium lactis*. These species are known for their effectiveness in preventing antibiotic-associated diarrhea [34], lowering risk of allergy [17] and supporting immune defence [35]. Since the potential health effects differ between probiotic species, the effects of one species cannot be directly generalized to all bacteria species. A systematic review concluded that healthy pregnant women tolerate probiotics well and their use in pregnancy is safe [36], further a randomized controlled trial reported no side effects to the newborn or mother [37].

When inspecting the participants’ responses to statements considering health effects of fish oil and probiotic supplements (based on timely evidence in literature), we observed that the responders were well aware of benefits of probiotics for the mother, while those for the unborn child less well. Approx. 45% of the participants from Finland erroneously considered that fish oil consumption leads to an increased risk of bleeding during delivery. It is of note that studies, including a scientific opinion by the European Food Safety Authority (EFSA), have demonstrated fish oil consumption up to 2.7 g/d during pregnancy safe [38]. Our results indicate that pregnant women have uncertainty about the possible health effects and safety of fish oil use during pregnancy, particularly in Finland. However, it should be noted that there are still knowledge gaps in research regarding the health benefits of the fish oil or probiotic supplements during pregnancy. We have shown before that health care professionals are the most common source of information for women regarding the use of vitamin and mineral food supplements during pregnancy [2]. Some country differences were observed as midwives and nurses were the primary source of information in Finland and in the UK, whilst doctors and general physicians in Italy and in Poland [2]. Similar findings have been seen in other studies too: nurses were identified as a primary source of information in Australian [39], and in US pregnant women [40]. In addition to health care personnel, women’s own intuition and governmental or ministerial web pages are important sources of information for pregnant women regarding the use of food supplements [2]. Further, the recommendation of the supplement use results in higher consumption of the food supplement in question, as appears to be the case for the use of fish oil supplements during pregnancy in Poland. However, it should be noted that further research is needed on whether the information sources differ according to food supplement type, i.e. fish oil or probiotics vs. vitamin and mineral supplements.

### Strengths and limitations

The strength of our study is the inclusion of participants from four different Western countries which are geographically and socioeconomically diverse and thus are anticipated to provide a comprehensive cross-sectional view on the use of fish oil and probiotics during pregnancy. Secondly, we collected product information about the food supplements used, from which we calculated the intakes of fish oil, and even more precisely about DHA and EPA, further we also specified the probiotic species, to get as much information as possible about the food supplement products used. The intake and frequency of use were the main focus in this study, while in many other studies, the information on the supplement consumption is reported as a secondary finding. In addition to product information, we collected extensive data on the participants using an electric questionnaire, and when preparing the study questionnaires, a translation process and successful pilot studies were conducted to ensure clarity of the questions as well as consistency among the countries [2].

Limitations include the need for additional recruitment methods in the UK, and, despite this, a lower sample size was gained in the UK as compared to the other countries. It should also be noted that 70% of the participants had a university level education, which might influence participants’ decisions to use dietary supplements. As it is generally known that the most highly educated individuals use nutritional supplements more frequently [33, 41], it can be assumed that the use of food supplements is less common in the entire pregnant population, highlighting the importance of health care professionals in counselling their patients in the appropriate use of food supplements. Further, food consumption habits and socio-economic status can possibly affect the participants’ decision-making regarding the use of food supplements, as studied before [26, 42], and for e.g., it has previously been reported that DHA intake is lower in low-income countries [8], and among those with lower socio-economic status [43]. In addition, we did not collect information about the women’s daily diet using a food diary, which could include e.g. fish oils in the form of fresh fish and foods containing probiotic bacteria, so the total daily intake, taking diet into account, has not been calculated.

## Conclusions

In conclusion, half of the pregnant women in the study used food supplements containing fish oil and one-tenth used supplements containing probiotics. Not all pregnant women were familiar with the prevailing recommendations for fish oil and probiotics supplement use, and further differences between countries were observed. We demonstrated that those women who use fish oil supplements receive the amount of DHA recommended by the researchers’ consensus, and that the use during pregnancy was more common in the country in which a recommendation for use during pregnancy has been issued i.e. Poland. Food supplements that contain probiotics were used the most in Finland. This study provides information on the current use of fish oil and probiotics during pregnancy, the knowledge regarding the recommendations as well as beliefs on the potential health benefits in four European countries, which should be considered in the health counselling to ensure appropriate use of the supplements to optimise maternal and neonatal health.

## Electronic supplementary material

Below is the link to the electronic supplementary material.

Supplementary Material 1

Supplementary Material 2

Supplementary Material 3

## References

[1] Food supplements| EFSA [WWW Document] (2024) URL https://www.efsa.europa.eu/en/topics/topic/food-supplements (accessed 4.25.24)

[2] Koivuniemi E, Hart K, Mazanowska N, Ruggeri S, Egan B, Censi L, Roccaldo R, Mattila L, Buonocore P, Löyttyniemi E, Raats MM, Wielgos M, Laitinen K (2022) Food supplement use differs from the recommendations in pregnant women: A multinational survey. Nutrients 14(14):2909. 10.3390/nu14142909PMID: 35889867; PMCID: PMC9322729 35889867 10.3390/nu14142909 PMC9322729

[3] WHO (2012) Guideline: daily iron and folic acid supplementation in pregnant women. Geneva, World Health Organization 23586119

[4] Saros L, Hart K, Koivuniemi E, Egan B, Raats M, Laitinen K (2024) Micronutrient supplement recommendations in pregnancy vary across a geographically diverse range of countries: a narrative review. Nutr Res 123:18–37 Epub 2023 Dec 26. PMID: 38228076 38228076 10.1016/j.nutres.2023.12.012

[5] Greenberg JA, Bell SJ, Ausdal WV Omega-3 fatty acid supplementation during pregnancy. Rev Obstet Gynecol 2008 Fall;1(4):162–169. PMID: 19173020; PMCID: PMC2621042. PMC2621042 19173020

[6] Burdge GC, Calder PC (2005) Sep-Oct;45(5):581– 97 Conversion of alpha-linolenic acid to longer-chain polyunsaturated fatty acids in human adults. Reprod Nutr Dev. 10.1051/rnd:2005047. PMID: 16188209 10.1051/rnd:2005047 16188209

[7] Gil-Sánchez A, Koletzko B, Larqué E (2012) Current understanding of placental fatty acid transport. Curr Opin Clin Nutr Metab Care.;15(3):265– 72. 10.1097/MCO.0b013e3283523b6e. PMID: 22450774 10.1097/MCO.0b013e3283523b6e 22450774

[8] Forsyth S, Gautier S, Salem N Jr (2016) Global estimates of dietary intake of docosahexaenoic acid and arachidonic acid in developing and developed countries. Ann Nutr Metab 68(4):258–267. 10.1159/000446855Epub 2016 Jun 9. PMID: 27288396 27288396 10.1159/000446855

[9] Commission Regulation (EU) No 440/2011 of 6 May 2011 on the authorisation and refusal of authorisation of certain health claims made on foods and referring to children’s development and healthText with EEA relevance, n.d

[10] Zimmer M, Sieroszewski P, Oszukowski P, Huras H, Fuchs T, Pawlosek A (2020) Polish society of gynecologists and obstetricians recommendations on supplementation during pregnancy. Ginekol Pol 91(10):644–653. 10.5603/GP.2020.0159 33184834 10.5603/GP.2020.0159

[11] Koletzko B, Cetin I, Thomas Brenna J (2007) Dietary fat intakes for pregnant and lactating women. Br J Nutr 98(5):873–877. 10.1017/S0007114507764747 17688705 10.1017/S0007114507764747

[12] Hill C, Guarner F, Reid G, Gibson GR, Merenstein DJ, Pot B, Morelli L, Canani RB, Flint HJ, Salminen S, Calder PC, Sanders ME (2014) Expert consensus document. The international scientific association for probiotics and prebiotics consensus statement on the scope and appropriate use of the term probiotic. Nat Rev Gastroenterol Hepatol 11(8):506–514. 10.1038/nrgastro.2014.66Epub 2014 Jun 10. PMID: 24912386 24912386 10.1038/nrgastro.2014.66

[13] Cuello-Garcia CA, Brożek JL, Fiocchi A, Pawankar R, Yepes-Nuñez JJ, Terracciano L, Gandhi S, Agarwal A, Zhang Y, Schünemann HJ (2015) Probiotics for the prevention of allergy: A systematic review and meta-analysis of randomized controlled trials. J Allergy Clin Immunol 136(4):952–961 Epub 2015 Jun 2. PMID: 26044853 26044853 10.1016/j.jaci.2015.04.031

[14] Rautava S, Kainonen E, Salminen S, Isolauri E (2012) Maternal probiotic supplementation during pregnancy and breast-feeding reduces the risk of eczema in the infant. J Allergy Clin Immunol.;130(6):1355-60. 10.1016/j.jaci.2012.09.003. Epub 2012 Oct 16. PMID: 23083673 10.1016/j.jaci.2012.09.003 23083673

[15] Wickens K, Black PN, Stanley TV, Mitchell E, Fitzharris P, Tannock GW, Purdie G, Crane J, Probiotic Study Group (2008) A differential effect of 2 probiotics in the prevention of eczema and atopy: a double-blind, randomized, placebo-controlled trial. J Allergy Clin Immunol 122(4):788–794 Epub 2008 Aug 31. PMID: 18762327 18762327 10.1016/j.jaci.2008.07.011

[16] Yu Q, Xu C, Wang M, Zhu J, Yu L, Yang Z, Liu S, Gao X (2022) The preventive and therapeutic effects of probiotics on mastitis: A systematic review and meta-analysis. PLoS ONE 17(9):e0274467. 10.1371/journal.pone.0274467PMID: 36084006; PMCID: PMC9462749 36084006 10.1371/journal.pone.0274467 PMC9462749

[17] Fiocchi A, Pawankar R, Cuello-Garcia C et al (2015) World allergy Organization-McMaster university guidelines for allergic disease prevention (GLAD-P): probiotics. World Allergy Organ J 8:1–13. 10.1186/s40413-015-0055-2 25628773 10.1186/s40413-015-0055-2 PMC4307749

[18] Knapik A, Kocot K, Witek A, Jankowski M, Wróblewska-Czech A, Kowalska M, Zejda JE, Brożek G (2018) Dietary supplementation usage by pregnant women in Silesia - population based study. Ginekol Pol.;89(9):506–512. 10.5603/GP.a2018.0086. PMID: 30318578 10.5603/GP.a2018.0086 30318578

[19] Gómez MF, Field CJ, Olstad DL, Loehr S, Ramage S, McCargar LJ, APrON Study Team (2015) Use of micronutrient supplements among pregnant women in Alberta: results from the Alberta pregnancy outcomes and nutrition (APrON) cohort. Matern Child Nutr 11(4):497–510. 10.1111/mcn.12038Epub 2013 Apr 5. PMID: 23557540; PMCID: PMC6860184 23557540 10.1111/mcn.12038 PMC6860184

[20] Shand AW, Walls M, Chatterjee R, Nassar N, Khambalia AZ (2016) Dietary vitamin, mineral and herbal supplement use: a cross-sectional survey of before and during pregnancy use in Sydney, Australia. Aust N Z J Obstet Gynaecol 56(2):154–161. 10.1111/ajo.12414Epub 2015 Oct 22. PMID: 26490392 26490392 10.1111/ajo.12414

[21] WHO Expert Committee on Physical Status (1995) The use and interpretation of anthropometry (‎1993: Geneva, S. Physical status: the use and interpretation of anthropometry. Report of a WHO expert committee. World Health Organ Tech Rep Ser 854:1–452. 10.1002/(sici)1520-6300(1996)8:6%3C786::aid-ajhb11%3E3.0.co;2-i 8594834

[22] Pahkala K, Heinonen OJ, Simell O, Viikari JSA, Rönnemaa T, Niinikoski H, Raitakari OT (2011) Association of physical activity with vascular endothelial function and Intima-Media thickness. Circulation 124:1956–1963. 10.1161/CIRCULATIONAHA.111.043851 21969011 10.1161/CIRCULATIONAHA.111.043851

[23] Olsen SF, Mikkelsen TB, Knudsen VK, Orozova-Bekkevold I, Halldórsson TI, Strøm M, Osterdal ML (2007) Data collected on maternal dietary exposures in the Danish National Birth Cohort. Paediatr Perinat Epidemiol.;21(1):76–86. 10.1111/j.1365-3016.2007.00777.x. PMID: 17239183 10.1111/j.1365-3016.2007.00777.x 17239183

[24] Thompson M, Hein N, Hanson C, Smith LM, Anderson-Berry A, Richter CK, Stessy Bisselou K, Kusi Appiah A, Kris-Etherton P, Skulas-Ray AC, Nordgren TM (2019) Omega-3 fatty acid intake by age, gender, and pregnancy status in the united States: National health and nutrition examination survey 2003⁻2014. Nutrients 11(1):177. 10.3390/nu11010177PMID: 30650613; PMCID: PMC6356780 30650613 10.3390/nu11010177 PMC6356780

[25] Gellert S, Schuchardt JP, Hahn A (2016) Higher omega-3 index and DHA status in pregnant women compared to lactating women - Results from a German nation-wide cross-sectional study. Prostaglandins Leukot Essent Fat Acids 109:22–28. 10.1016/j.plefa.2016.04.002Epub 2016 Apr 19. PMID: 27269710 10.1016/j.plefa.2016.04.002 27269710

[26] Bärebring L, Mullally D, Glantz A, Elllis J, Hulthén L, Jagner Å, Bullarbo M, Winkvist A, Augustin H (2018) Sociodemographic factors associated with dietary supplement use in early pregnancy in a Swedish cohort. Br J Nutr 119(1):90–95 Epub 2017 Dec 4. PMID: 29198190 29198190 10.1017/S0007114517003270

[27] Oliver EM, Grimshaw KE, Schoemaker AA, Keil T, McBride D, Sprikkelman AB, Ragnarsdottir HS, Trendelenburg V, Emmanouil E, Reche M, Fiocchi A, Fiandor A, Stanczyk-Przyluska A, Wilczynski J, Busacca M, Sigurdardottir ST, Dubakiene R, Rudzeviciene O, Vlaxos GD, Beyer K, Roberts G (2014) Dietary habits and supplement use in relation to national pregnancy recommendations: data from the EuroPrevall birth cohort. Matern Child Health J.;18(10):2408-25. 10.1007/s10995-014-1480-5. PMID: 24752313 10.1007/s10995-014-1480-5 24752313

[28] Moran-Lev H, Bauer S, Farhi A, Nehama H, Yerushalmy-Feler A, Mandel D, Lubetzky R (2019) Nutrition and the use of supplements in women during pregnancy: A Cross-Sectional survey. Food Nutr Bull 40(2):231–240 Epub 2019 May 9. PMID: 31072212 31072212 10.1177/0379572119833857

[29] McAlpine JM, Vanderlelie JJ, Vincze LJ, Perkins AV (2020) Use of micronutrient supplements in pregnant women of south-east Queensland. Aust N Z J Obstet Gynaecol 60(4):561–567. 10.1111/ajo.13109Epub 2020 Jan 5. PMID: 31903555 31903555 10.1111/ajo.13109

[30] Pouchieu C, Lévy R, Faure C, Andreeva VA, Galan P, Hercberg S, Touvier M (2013) Socioeconomic, lifestyle and dietary factors associated with dietary supplement use during pregnancy. PLoS ONE 8(8):e70733. 10.1371/journal.pone.0070733PMID: 23967094; PMCID: PMC3742608 23967094 10.1371/journal.pone.0070733 PMC3742608

[31] Jun S, Gahche JJ, Potischman N, Dwyer JT, Guenther PM, Sauder KA, Bailey RL (2020) Dietary Supplement Use and Its Micronutrient Contribution During Pregnancy and Lactation in the United States. Obstet Gynecol.;135(3):623–633. doi: 10.1097/AOG.0000000000003657. Erratum in: Obstet Gynecol. 2020;135(6):1489. PMID: 32028492; PMCID: PMC7138460 10.1097/AOG.0000000000003657 PMC7138460 32028492

[32] Wierzejska R, Jarosz M, Wojda B, Siuba-Strzelińska M (2018) Dietary intake of DHA during pregnancy: a significant gap between the actual intake and current nutritional recommendations. Rocz Panstw Zakl Hig.;69(4):381–386. 10.32394/rpzh.2018.0044. PMID: 30525329 10.32394/rpzh.2018.0044 30525329

[33] Arkkola T, Uusitalo U, Pietikäinen M, Metsälä J, Kronberg-Kippilä C, Erkkola M, Veijola R, Knip M, Virtanen SM, Ovaskainen ML (2006) Dietary intake and use of dietary supplements in relation to demographic variables among pregnant Finnish women. Br J Nutr.;96(5):913– 20. 10.1017/bjn20061929. PMID: 17092382 10.1017/bjn20061929 17092382

[34] Szajewska H, Canani RB, Guarino A, Hojsak I, Indrio F, Kolacek S, Orel R, Shamir R, Vandenplas Y, van Goudoever JB, Weizman Z (2016) ESPGHAN Working Group for ProbioticsPrebiotics. Probiotics for the Prevention of Antibiotic-Associated Diarrhea in Children. J Pediatr Gastroenterol Nutr.;62(3):495–506. 10.1097/MPG.0000000000001081. PMID: 26756877 10.1097/MPG.0000000000001081 26756877

[35] Jungersen M, Wind A, Johansen E, Christensen JE, Stuer-Lauridsen B, Eskesen D (2014) The science behind the probiotic strain bifidobacterium animalis subsp. Lactis BB-12(^®^). Microorganisms 2(2):92–110. 10.3390/microorganisms2020092PMID: 27682233; PMCID: PMC5029483 27682233 10.3390/microorganisms2020092 PMC5029483

[36] Navarro-Tapia E, Sebastiani G, Sailer S, Toledano LA, Serra-Delgado M, García-Algar Ó, Andreu-Fernández V (2020) Probiotic supplementation during the perinatal and infant period: effects on gut dysbiosis and disease. Nutrients 12(8):2243. 10.3390/nu12082243PMID: 32727119; PMCID: PMC7468726 32727119 10.3390/nu12082243 PMC7468726

[37] Baldassarre ME, Di Mauro A, Mastromarino P, Fanelli M, Martinelli D, Urbano F, Capobianco D, Laforgia N (2016) Administration of a Multi-Strain probiotic product to women in the perinatal period differentially affects the breast milk cytokine profile and May have beneficial effects on neonatal Gastrointestinal functional symptoms. A randomized clinical trial. Nutrients 8(11):677. 10.3390/nu8110677PMID: 27801789; PMCID: PMC5133065 27801789 10.3390/nu8110677 PMC5133065

[38] Scientific Opinion on the Tolerable Upper (2012) Intake level of eicosapentaenoic acid (EPA), docosahexanoic acid (DHA) and docosapentaenoic acid (DHA). EFSA J 10(7):2815 10.2903/j.efsa.2012.2815 PMC13093066 42016086

[39] Barnes LAJ, Barclay L, McCaffery K et al (2019) Factors influencing women’s decision-making regarding complementary medicine product use in pregnancy and lactation. BMC Pregnancy Childbirth 19:280. 10.1186/s12884-019-2396-2 31390996 10.1186/s12884-019-2396-2 PMC6686446

[40] Jun SMPH, Gahche JJ, PhD MPH, Potischman NPD, Dwyer JT, DSc RD, Guenther PM, PhD RD, Sauder KAPD, Bailey RL, PhD MPH, RD. Dietary Supplement Use and Its Micronutrient Contribution During Pregnancy and Lactation in the United States. Obstetrics & Gynecology 135(3):p 623–633, March 2020.| 10.1097/AOG.0000000000003657 10.1097/AOG.0000000000003657 PMC7138460 32028492

[41] Aronsson C, Vehik K, Yang J, Uusitalo U, Hay K, Joslowski G, Norris J (2013) Use of dietary supplements in pregnant women in relation to sociodemographic factors – a report from the environmental determinants of diabetes in the young (TEDDY) study. Public Health Nutr 16(8):1390–1402. 10.1017/S1368980013000293 23452986 10.1017/S1368980013000293 PMC4112516

[42] Camier A, Kadawathagedara M, Lioret S, Bois C, Cheminat M, Dufourg MN, Charles MA, de Lauzon-Guillain B (2019) Social inequalities in prenatal folic acid supplementation: results from the ELFE cohort. Nutrients 11(5):1108. 10.3390/nu11051108PMID: 31109064; PMCID: PMC6566921 31109064 10.3390/nu11051108 PMC6566921

[43] Nochera CL, Goossen LH, Brutus AR, Cristales M, Eastman B (2011) Consumption of DHA + EPA by low-income women during pregnancy and lactation. Nutr Clin Pract.;26(4):445– 50. 10.1177/0884533611406133. Epub 2011 Jul 1. PMID: 21724916 10.1177/0884533611406133 21724916

