# Supplementary material for: Fish oil and probiotic food supplements: consumptions and attitudes of pregnant women in four European countries
Source: Eur J Nutr. 2025 Apr 5;64(4):146. doi: 10.1007/s00394-025-03654-5 (PMC11972203; doi:10.1007/s00394-025-03654-5)
Supplement: Supplementary file 1 — Supplementary Material 1 [file 394_2025_3654_MOESM1_ESM.pdf]

European Journal of Nutrition

Online resource 1

Fish oil and probiotic food supplements: Consumptions and attitudes of pregnant women in four European countries

Kristiina Jaakkola[1], Ella Koivuniemi[1,2], Kathryn Hart[3], Natalia Mazanowska[4], Romana Roccaldo[5], Laura Censi[5], Bernadette Egan[3], Lilja Mattila[1], Pasquale Buonocore[5], Eliisa Löyttyniemi[6], Monique Raats[3], Stefania Ruggeri[5], Miroslaw Wielgos[7,8], Kirsi Laitinen[1,2]

Affiliations

[1]Research Centre for Integrative Physiology and Pharmacology, Institute of Biomedicine, Faculty of Medicine, University of Turku, Turku, Finland

[2]Nutrition and Food Research Center, Faculty of Medicine, University of Turku, Turku, Finland

[3] Department of Nutritional Sciences, School of Biosciences and Medicine, Faculty of Health and Medical Sciences, University of Surrey, Guildford, UK

[4] Department of Obstetrics and Gynecology, Institute of Mother and Child, Warsaw, Poland

[5]Council for Agricultural Research and Economics (CREA), Research Centre for Food and Nutrition, Rome, Italy

[6]Biostatistics, Department of Clinical Medicine, University of Turku, Turku, Finland

[7]Department of Obstetrics and Perinatology, National Medical Institute of the Ministry of Interior and Administration, Warsaw, Poland

[8]Medical Faculty, Lazarski University, Warsaw, Poland

ORCID IDs:

Ella Koivuniemi https://orcid.org/0000-0002-5751-3064

Kathryn Hart https://orcid.org/0000-0001-7494-9866

Natalia Mazanowska https://orcid.org/0000-0002-6970-5303

Romana Roccaldo https://orcid.org/0000-0003-2976-3864

Laura Censi https://orcid.org/0000-0002-8769-6914

Bernadette Egan https://orcid.org/0000-0002-5767-0457

Eliisa Löyttyniemi https://orcid.org/0000-0002-7278-6511

Monique Raats https://orcid.org/0000-0002-8057-2783

Miroslaw Wielgos https://orcid.org/0000-0003-2581-3668

Kirsi Laitinen https://orcid.org/0000-0001-5245-8118

Corresponding author:

Kirsi Laitinen

Nutrition and Food Research Center & Institute of Biomedicine

Faculty of Medicine

University of Turku

Turku
Finland
kirsi.laitinen@utu.fi

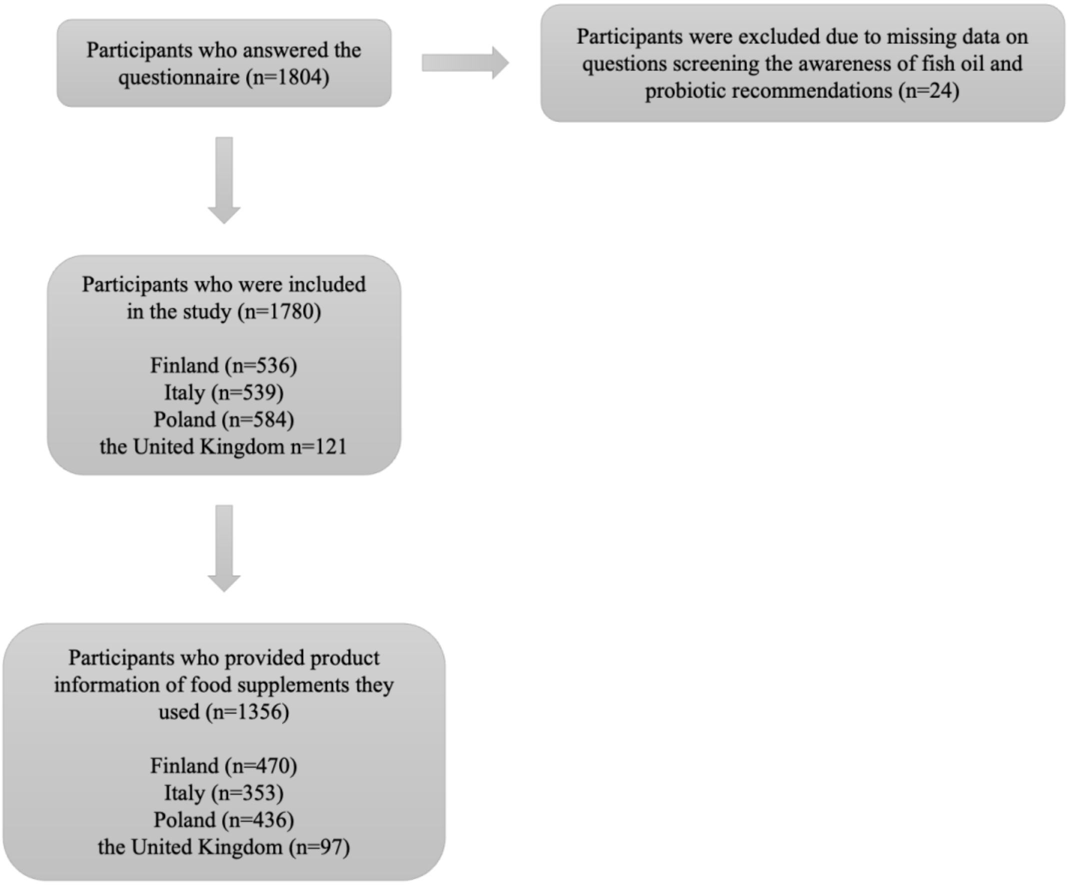

Online resource 1. Flow chart of the study.
