# Supplementary material for: Fish oil and probiotic food supplements: consumptions and attitudes of pregnant women in four European countries
Source: Eur J Nutr. 2025 Apr 5;64(4):146. doi: 10.1007/s00394-025-03654-5 (PMC11972203; doi:10.1007/s00394-025-03654-5)
Supplement: Supplementary file 2 — Supplementary Material 2 [file 394_2025_3654_MOESM2_ESM.pdf]

European Journal of Nutrition

Online resource 2

Fish oil and probiotic food supplements: Consumptions and attitudes of pregnant women in four European countries

Kristiina Jaakkola[1], Ella Koivuniemi[1,2], Kathryn Hart[3], Natalia Mazanowska[4], Romana Roccaldo[5], Laura Censi[5], Bernadette Egan[3], Lilja Mattila[1], Pasquale Buonocore[5], Eliisa Löyttyniemi[6], Monique Raats[3], Stefania Ruggeri[5], Miroslaw Wielgos[7,8], Kirsi Laitinen[1,2]

Affiliations

[1]Research Centre for Integrative Physiology and Pharmacology, Institute of Biomedicine, Faculty of Medicine, University of Turku, Turku, Finland

[2]Nutrition and Food Research Center, Faculty of Medicine, University of Turku, Turku, Finland

[3] Department of Nutritional Sciences, School of Biosciences and Medicine, Faculty of Health and Medical Sciences, University of Surrey, Guildford, UK

[4] Department of Obstetrics and Gynecology, Institute of Mother and Child, Warsaw, Poland

[5]Council for Agricultural Research and Economics (CREA), Research Centre for Food and Nutrition, Rome, Italy

[6]Biostatistics, Department of Clinical Medicine, University of Turku, Turku, Finland

[7]Department of Obstetrics and Perinatology, National Medical Institute of the Ministry of Interior and Administration, Warsaw, Poland

[8]Medical Faculty, Lazarski University, Warsaw, Poland

ORCID IDs:

Ella Koivuniemi https://orcid.org/0000-0002-5751-3064

Kathryn Hart https://orcid.org/0000-0001-7494-9866

Natalia Mazanowska https://orcid.org/0000-0002-6970-5303

Romana Roccaldo https://orcid.org/0000-0003-2976-3864

Laura Censi https://orcid.org/0000-0002-8769-6914

Bernadette Egan https://orcid.org/0000-0002-5767-0457

Eliisa Löyttyniemi https://orcid.org/0000-0002-7278-6511

Monique Raats https://orcid.org/0000-0002-8057-2783

Miroslaw Wielgos https://orcid.org/0000-0003-2581-3668

Kirsi Laitinen https://orcid.org/0000-0001-5245-8118

Corresponding author:

Kirsi Laitinen

Nutrition and Food Research Center & Institute of Biomedicine

Faculty of Medicine

University of Turku

Turku
Finland
kirsi.laitinen@utu.fi

Online resource 2. Morbidity of the participants by country (n=1780).

	Total n	All	Finland	Italy	Poland	United Kingdom
		n (%)				
**Medical conditions associated with this pregnancy**	1776/536/538/581/121					
Gestational diabetes		142 (8.0)	62 (11.6)	25 (4.6)	52 (9.0)	3 (2.5)
Pre-eclampsia		6 (0.3)	1 (0.2)	4 (0.7)	1 (0.2)	0 (0.0)
Hypertension		24 (1.4)	7 (1.3)	6 (1.1)	11 (1.9)	0 (0.0)
Pregnancy related nausea		628 (35.4)	213 (39.7)	206 (38.3)	157 (27.0)	52 (43.0)
**Medical conditions**	1747/536/528/565/118					
Overweight/obesity		336 (19.2)	121 (22.6)	103 (19.5)	93 (16.5)	19 (16.1)
Type I diabetes		8 (0.5)	5 (0.9)	2 (0.4)	1 (0.2)	0 (0.0)
Type II diabetes		9 (0.5)	2 (0.4)	3 (0.6)	4 (0.7)	0 (0.0)
Type I AND/OR type II diabetes		17 (1.0)	7 (1.3)	5 (0.9)	5 (0.9)	0 (0.0)
Heart disease/ hypertension/ high cholesterol		38 (2.2)	18 (3.4)	6 (1.1)	13 (2.3)	1 (0.8)
Thyroid disease		273 (15.6)	52 (9.7)	70 (13.3)	141 (25.0)	10 (8.5)
Celiac disease		11 (0.6)	4 (0.7)	4 (0.8)	2 (0.4)	1 (0.8)
Inflammatory bowel diseases		13 (0.7)	4 (0.7)	7 (1.3)	2 (0.4)	0 (0.0)
Irritable bowel syndrome		127 (7.3)	42 (7.8)	37 (7.0)	29 (5.1)	19 (16.1)
Food intolerance		111 (6.4)	44 (8.2)	42 (8.0)	16 (2.8)	9 (7.6)
Celiac disease, IBS[a], IBD[b], and/or food intolerance		220 (12.6)	81 (15.1)	72 (13.6)	43 (7.6)	24 (20.3)
Allergy		291 (16.7)	149 (27.8)	57 (10.8)	78 (13.8)	7 (5.9)
Asthma		126 (7.2)	58 (10.8)	27 (5.1)	28 (5.0)	13 (11.0)
Rheumatism, arthritis		29 (1.7)	5 (0.9)	12 (2.3)	9 (1.6)	3 (2.5)
Migraine or frequent headaches		270 (15.5)	119 (22.2)	50 (9.5)	89 (15.8)	12 (10.2)
Chronic anxiety or depression		105 (6.0)	49 (9.1)	25 (4.7)	16 (2.8)	15 (12.7)
Other health problems		155 (8.9)	53 (9.9)	33 (6.3)	60 (10.6)	9 (7.6)

[a]Irritable bowel syndrome

[b]Inflammatory bowel diseases (Crohn’s disease, Ulcerative colitis)
