# Supplementary material for: Fish oil and probiotic food supplements: consumptions and attitudes of pregnant women in four European countries
Source: Eur J Nutr. 2025 Apr 5;64(4):146. doi: 10.1007/s00394-025-03654-5 (PMC11972203; doi:10.1007/s00394-025-03654-5)
Supplement: Supplementary file 3 — Supplementary Material 3 [file 394_2025_3654_MOESM3_ESM.pdf]

European Journal of Nutrition
Online resource 3

Fish oil and probiotic food supplements: Consumptions and attitudes of pregnant women in four European countries

Kristiina Jaakkola[1], Ella Koivuniemi[1,2], Kathryn Hart[3], Natalia Mazanowska[4], Romana Roccaldo[5], Laura Censi[5], Bernadette Egan[3], Lilja Mattila[1], Pasquale Buonocore[5], Eliisa Löyttyniemi[6], Monique Raats[3], Stefania Ruggeri[5], Miroslaw Wielgos[7,8], Kirsi Laitinen[1,2]

Affiliations
[1]Research Centre for Integrative Physiology and Pharmacology, Institute of Biomedicine, Faculty of Medicine, University of Turku, Turku, Finland
[2]Nutrition and Food Research Center, Faculty of Medicine, University of Turku, Turku, Finland
[3] Department of Nutritional Sciences, School of Biosciences and Medicine, Faculty of Health and Medical Sciences, University of Surrey, Guildford, UK
[4] Department of Obstetrics and Gynecology, Institute of Mother and Child, Warsaw, Poland
[5]Council for Agricultural Research and Economics (CREA), Research Centre for Food and Nutrition, Rome, Italy
[6]Biostatistics, Department of Clinical Medicine, University of Turku, Turku, Finland
[7]Department of Obstetrics and Perinatology, National Medical Institute of the Ministry of Interior and Administration, Warsaw, Poland
[8]Medical Faculty, Lazarski University, Warsaw, Poland

ORCID IDs:
Ella Koivuniemi https://orcid.org/0000-0002-5751-3064
Kathryn Hart https://orcid.org/0000-0001-7494-9866
Natalia Mazanowska https://orcid.org/0000-0002-6970-5303
Romana Roccaldo https://orcid.org/0000-0003-2976-3864
Laura Censi https://orcid.org/0000-0002-8769-6914
Bernadette Egan https://orcid.org/0000-0002-5767-0457
Eliisa Löyttyniemi https://orcid.org/0000-0002-7278-6511
Monique Raats https://orcid.org/0000-0002-8057-2783
Miroslaw Wielgos https://orcid.org/0000-0003-2581-3668
Kirsi Laitinen https://orcid.org/0000-0001-5245-8118

Corresponding author:
Kirsi Laitinen
Nutrition and Food Research Center & Institute of Biomedicine
Faculty of Medicine
University of Turku

Turku
Finland
kirsi.laitinen@utu.fi

Online resource 3. Number of users of specific food supplements and probiotic species from participant-reported product information by country (n=1356).

	Total n	All	Finland	Italy	Poland	United Kingdom
		n (%)				
DHA	1356/470/353/436/97	315 (23.2)	8 (1.7)	123 (34.8)	182 (41.7)	2 (2.1)
EPA	1356/470/353/436/97	3 (0.2)	3 (0.6)	0 (0.0)	0 (0.0)	0 (0.0)
DHA+EPA	1356/470/353/436/97	352 (26.0)	85 (18.1)	82 (23.2)	154 (35.3)	31 (32.0)
Probiotics[a]	1356/470/353/436/97	132 (9.7)	85 (18.1)	27 (7.6)	11 (2.5)	9 (9.3)
Lacticaseibacillus rhamnosus	1356/470/353/436/97	87 (6.4)	70 (14.9)	8 (2.3)	6 (1.4)	3 (3.1)
L. bulgaricus	1356/470/353/436/97	12 (0.9)	8 (1.7)	2 (0.6)	1 (0.2)	1 (1.0)
L. sporogenes	1356/470/353/436/97	1 (0.1)	1 (0.2)	0 (0.0)	0 (0.0)	0 (0.0)
L. acidophilus	1356/470/353/436/97	57 (4.2)	44 (9.4)	5 (1.4)	3 (0.7)	5 (5.2)
Bifidobacterium bifidum	1356/470/353/436/97	22 (1.6)	14 (3.0)	3 (0.8)	1 (0.2)	4 (4.1)
Streptococcus thermophilus	1356/470/353/436/97	28 (2.1)	20 (4.3)	3 (0.8)	2 (0.5)	3 (3.1)
L. plantarum	1356/470/353/436/97	27 (2.0)	18 (3.8)	3 (0.8)	6 (1.4)	0 (0.0)
Bifidobacterium lactis	1356/470/353/436/97	66 (4.9)	47 (10.0)	12 (3.4)	2 (0.5)	5 (5.2)
L. reuteri	1356/470/353/436/97	27 (2.0)	25 (5.3)	0 (0.0)	0 (0.0)	2 (2.1)
L. casei	1356/470/353/436/97	38 (2.8)	30 (6.4)	5 (1.4)	2 (0.5)	1 (1.0)
L. paracasei	1356/470/353/436/97	23 (1.7)	15 (3.2)	4 (1.1)	1 (0.2)	3 (3.1)
Bifidobacterium longum	1356/470/353/436/97	28 (2.1)	23 (4.9)	3 (0.8)	2 (0.5)	0 (0.0)
Bifidobacterium breve	1356/470/353/436/97	14 (1.0)	9 (1.9)	4 (1.1)	1 (0.2)	0 (0.0)
Bifidobacterium infantis	1356/470/353/436/97	5 (0.4)	3 (0.6)	1 (0.3)	0 (0.0)	1 (1.0)
L. brevis	1356/470/353/436/97	2 (0.1)	2 (0.4)	0 (0.0)	0 (0.0)	0 (0.0)
L. fermentum	1356/470/353/436/97	6 (0.4)	2 (0.4)	2 (0.6)	2 (0.5)	0 (0.0)
Bacillus coagulans	1356/470/353/436/97	14 (1.0)	4 (0.9)	10 (2.8)	0 (0.0)	0 (0.0)
L. helveticus spp. jugurti	1356/470/353/436/97	4 (0.3)	2 (0.4)	0 (0.0)	2 (0.5)	0 (0.0)
Lactococcus lactis	1356/470/353/436/97	5 (0.4)	2 (0.4)	3 (0.8)	0 (0.0)	0 (0.0)

Enterococcus faecium	1356/470/353/436/97	5 (0.4)	1 (0.2)	4 (1.1)	0 (0.0)	0 (0.0)
Lactobacillus salivarius	1356/470/353/436/97	4 (0.3)	1 (0.2)	0 (0.0)	0 (0.0)	3 (3.1)

[a]The top three most used probiotic species in Finland: *Lacticaseibacillus rhamnosus* (formerly *Lactobacillus rhamnosus*), *Bifidobacterium lactis*, *L. acidophilus*, in Italy: *Bifidobacterium lactis*, *Bacillus* coagulans, *Lacticaseibacillus rhamnosus* (formerly *Lactobacillus rhamnosus*), in Poland: *Lacticaseibacillus rhamnosus* (formerly *Lactobacillus rhamnosus*), *L. plantarum*, *L. acidophilus*, and in UK: *L. acidophilus*, *Bifidobacterium lactis*, *Bifidobacterium bifidum*.
